# Infectious Acute Pancreatitis: Salmonella Typhimurium

**DOI:** 10.7759/cureus.114848

**Published:** 2026-08-20

**Authors:** Preskila Gurung, Julius Nwajei, Uchechukwu Obiagwu, David Umeana, Bassam Fallouh

**Affiliations:** 1 Acute Medicine, William Harvey Hospital, Ashford, GBR; 2 General Medicine, William Harvey Hospital, Ashford, GBR

**Keywords:** acute pancreatitis, gastroenteritis, non-typhoidal salmonella (nts), salmonella gastroenteritis, salmonella infection

## Abstract

Acute pancreatitis is most commonly attributed to gallstones or alcohol consumption. Infectious etiologies remain rare and are often overlooked. *Salmonella enterica* typically presents as self-limiting gastroenteritis, but it can occasionally lead to severe systemic complications. We report a case precipitated by *S. enterica* serotype Typhimurium in a 48-year-old male returning from North Africa.

History-taking, physical examination, and investigations ruled out common biliary and metabolic causes and confirmed *Salmonella *infection. The patient was treated with intravenous antibiotics and supportive care, leading to significant clinical and biochemical improvement.

This case highlights the importance of clinicians maintaining a high index of suspicion for infectious causes of pancreatitis, particularly in patients with a relevant clinical history.

## Introduction

Acute pancreatitis is an acute inflammatory process of the pancreas with varying involvement of local tissues or more remote organ systems. Symptoms may range from mild pancreatitis, which is the most common form, to severe pancreatitis, which is characterized by persistent single- or multiorgan failure for more than 48 hours [1].

In the United Kingdom (UK), the most common cause of acute pancreatitis is gallstones, accounting for approximately 50% of cases, while alcohol is the second most common cause, accounting for approximately 25% of cases [2]. Other causes include postendoscopic procedures, such as endoscopic retrograde cholangiopancreatography (ERCP); metabolic conditions, such as hypertriglyceridemia and hypercalcemia; and drugs [2]. Approximately 10% of cases of acute pancreatitis are thought to be of infectious origin, including viral, bacterial, and parasitic infections [3].

*Salmonella *is a foodborne pathogen that causes human gastroenteritis. Globally, *Salmonella *is transmitted through the consumption of contaminated food and water. The World Health Organization (WHO) describes *Salmonella *as one of the four most important causes of diarrhea worldwide [4]. In the UK, *Salmonella *infections have been steeply increasing, with a 17.1% increase from 2023, from 8,872 cases in 2023 to 10,388 cases in 2024. Children younger than 10 years were particularly affected, accounting for 21.5% of cases [5].

Approximately 15% of infected individuals in areas endemic for *Salmonella *infection experience gastrointestinal complications such as pancreatitis, hepatitis, and cholecystitis [4]. The relationship between *Salmonella *infection and acute pancreatitis is rarely observed, with very few documented cases in the literature. Despite its low incidence, *Salmonella*-associated pancreatitis usually presents as biochemical pancreatitis, with elevated serum amylase and lipase levels in the absence of clinical features such as epigastric pain and vomiting [6]. It is important to differentiate biochemical pancreatitis from clinical pancreatitis, as untreated clinical pancreatitis can lead to complications such as necrotizing pancreatitis.

In this case report, we describe the clinical presentation, diagnostic dilemma, management strategies, and treatment outcome of a case of acute pancreatitis induced by *Salmonella *infection in a middle-aged man from the UK who had recently returned from a holiday in a North African country.

## Case presentation

A 48-year-old man presented to the hospital with a five-day history of fever, followed the next day by diarrhea, nausea, and recurrent bilious vomiting. These symptoms were associated with abdominal pain and cramps that later localized to the epigastric region. His symptoms developed approximately seven days after returning from a recent holiday in North Africa. He reported eight to nine episodes of diarrhea mixed with mucus and blood, along with reduced oral intake due to persistent vomiting. He also reported consuming seafood during the holiday.

His past medical history included Lynch syndrome and a family history of colon cancer, but he was otherwise well. He had no history of pancreatic or significant liver disease or alcohol abuse and had not consumed alcohol in the month preceding presentation. He had not started any new prescription, herbal, or over-the-counter medications. Aspirin was his only regular medication.

On admission, he was hemodynamically stable and afebrile, with ongoing nausea and vomiting. Physical examination revealed slight abdominal guarding, with tenderness in the lower quadrant and epigastric region and hypoactive bowel sounds. He was moderately dehydrated, but examination of other systems was unremarkable.

Laboratory investigations showed normal renal and liver function and electrolyte levels, with a slight elevation in C-reactive protein (27 mg/L; normal range, 0-10 mg/L) and a normal white blood cell count (6.2 × 10^9^/L; normal range, 4-11 × 10^9^/L). Venous blood gas analysis showed a normal pH (7.37) and a slightly elevated lactate level (2.3 mmol/L; normal range, 0.0-1.6 mmol/L). Amylase was elevated at 379 U/L (reference range, 0-125 U/L). Blood and stool cultures were obtained within 24 hours of admission. Table 1 summarizes the laboratory results at presentation.

**Table 1:** Laboratory results on admission

Test	Result	Reference range
C-reactive protein	27 mg/L	0-10 mg/L
Hemoglobin	149 g/L	130-180 g/L
White blood cells	6.2 x 10^9^/L	4-11 x 10^9^/L
Platelets	220 x 10^9^/L	150-400 x 10^9^/L
Neutrophils	5.4 x 10^9^/L	2-7.5 x 10^9^/L
Lymphocytes	0.6 x 10^9^/L	1.5-4 x 10^9^/L
Amylase	379 U/L	0-125 U/L

The initial working diagnosis on admission was acute infectious gastroenteritis based on the patient’s symptoms of diarrhea and fever and his recent travel history. Initial management was therefore conservative, with intravenous fluids, antiemetics, intravenous antibiotics (amoxicillin-clavulanate), and analgesia.

Serum amylase was elevated, which was initially attributed to the ongoing gastroenteritis rather than pancreatitis during the early stage of hospitalization. However, given the elevated amylase level, severity of the patient’s symptoms, and history of Lynch syndrome, CT of the abdomen and pelvis (CT AP) was requested to assess for gastrointestinal complications and exclude alternative intra-abdominal pathology, such as colon cancer.

**Figure 1:**
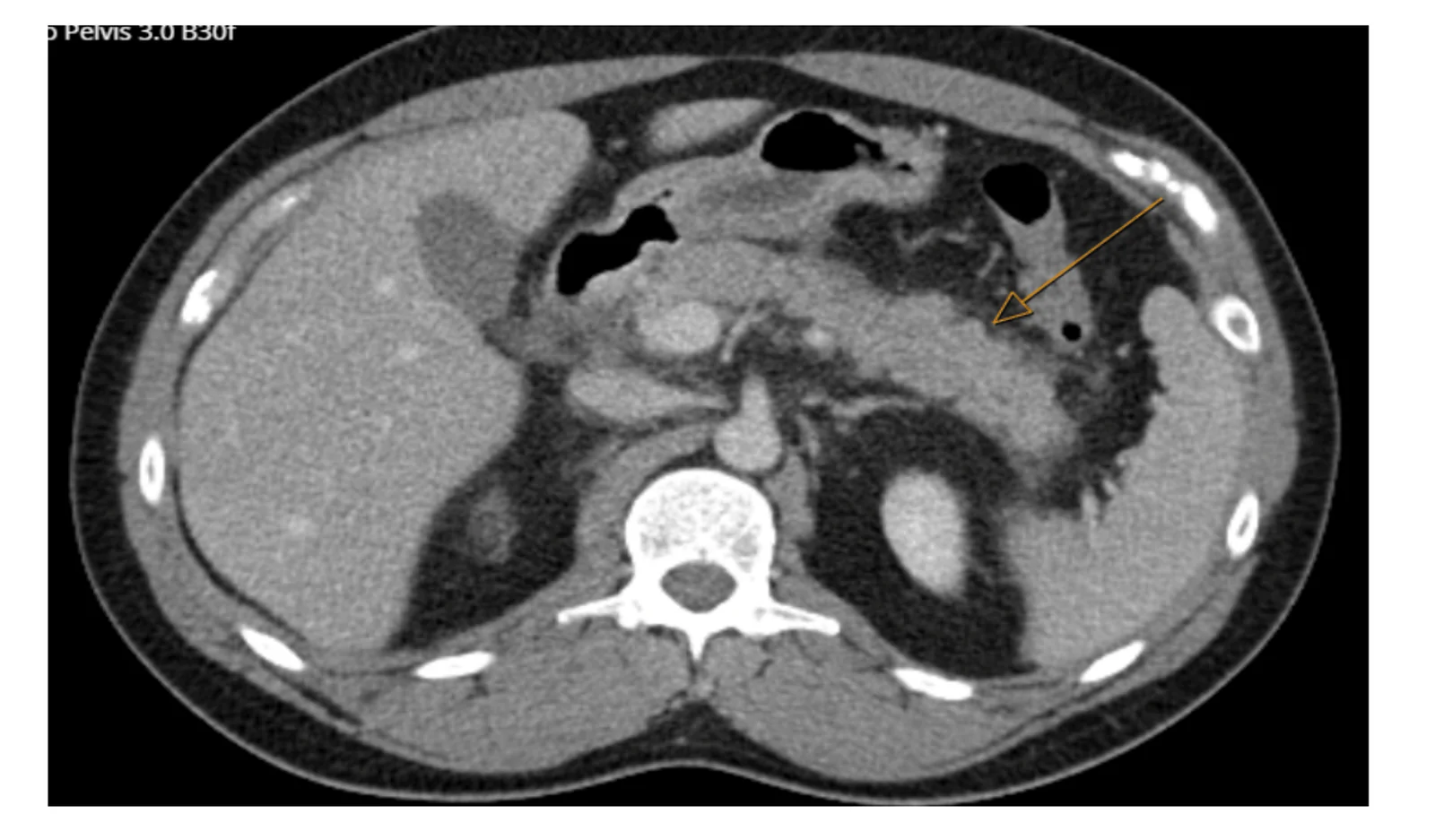
Axial contrast-enhanced CT of the abdomen and pelvis showing an enlarged, inflamed pancreas with surrounding fat stranding, consistent with acute pancreatitis (arrow)

**Figure 2:**
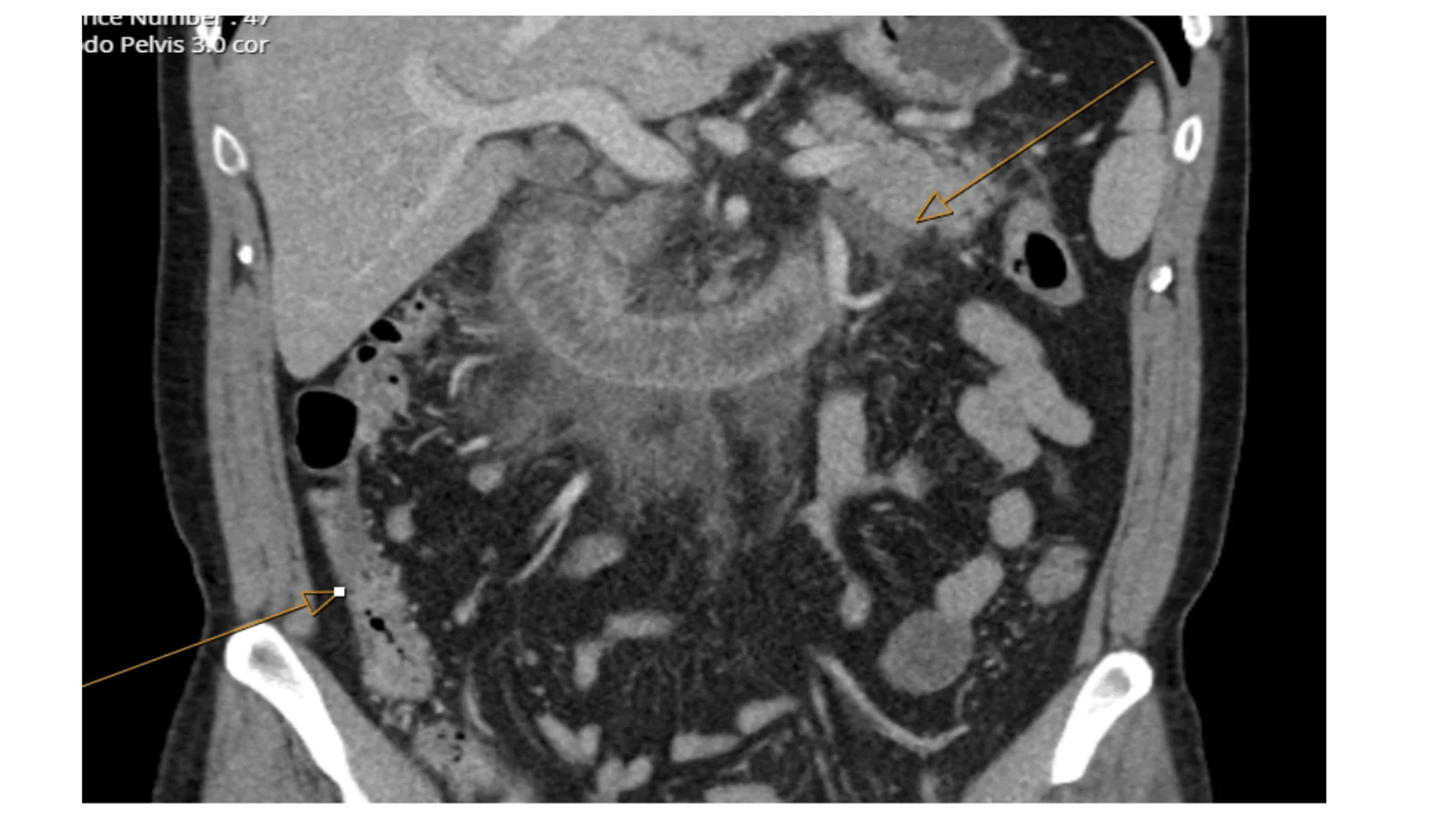
Coronal contrast-enhanced CT of the abdomen and pelvis showing normal wall thickness of the ascending colon (lower arrow) and features of acute pancreatitis with adjacent inflammation of the duodenum (upper arrow)

CT showed features consistent with acute pancreatitis (Figures 1, 2). Given the clinical presentation, elevated amylase level, and radiological confirmation of pancreatitis, a diagnosis of acute pancreatitis was made.

A range of investigations, including ultrasound, CT, MRI, further blood tests, and stool culture, were subsequently performed to determine the underlying cause. CT imaging and stool culture were particularly important in establishing the diagnosis, with the second CT of the abdomen and pelvis confirming colitis with ongoing pancreatitis. These investigations enabled a clinical correlation among severe *Salmonella *gastroenteritis, associated colitis, and pancreatitis.

Various imaging studies were performed to rule out gallstones as the cause of pancreatitis. Dedicated blood tests were also conducted to exclude other causes, including a lipid profile (triglyceride), which was unremarkable. An autoimmune screen, including antinuclear antibodies (ANA), antineutrophil cytoplasmic antibodies (ANCA), immunoglobulins, including IgG, and electrophoresis, was unremarkable apart from evidence of an acute-phase reaction. Calcium levels were within normal limits. Tumor markers, including CA 19-9 and alpha-fetoprotein, were also unremarkable.

Detailed history-taking during the early stages of hospitalization was crucial in ruling out other potential etiologies of pancreatitis, including alcohol use, trauma, medications, drugs, and recent procedures such as ERCP. Despite the extensive workup, the cause of the pancreatitis remained unclear.

**Figure 3:**
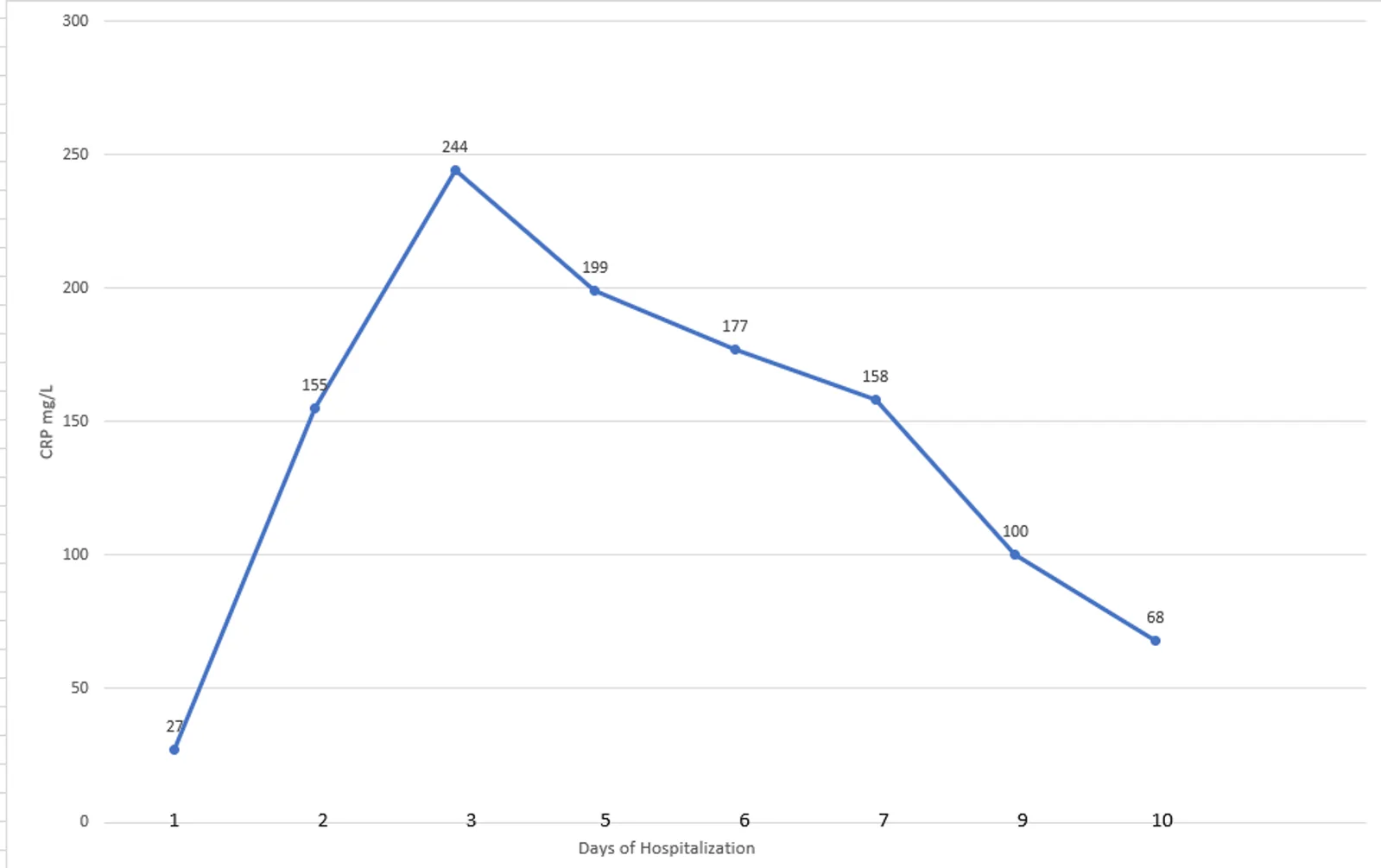
Trend in C-reactive protein during hospitalization

However, on Day 2 of hospitalization, his CRP increased from 27 to 155 mg/L, reaching a peak of 244 mg/L on Day 3 (Figure 3). This increase correlated with a change in his clinical condition, evidenced by a new spike in temperature of 38.4°C and tachycardia.

**Figure 4:**
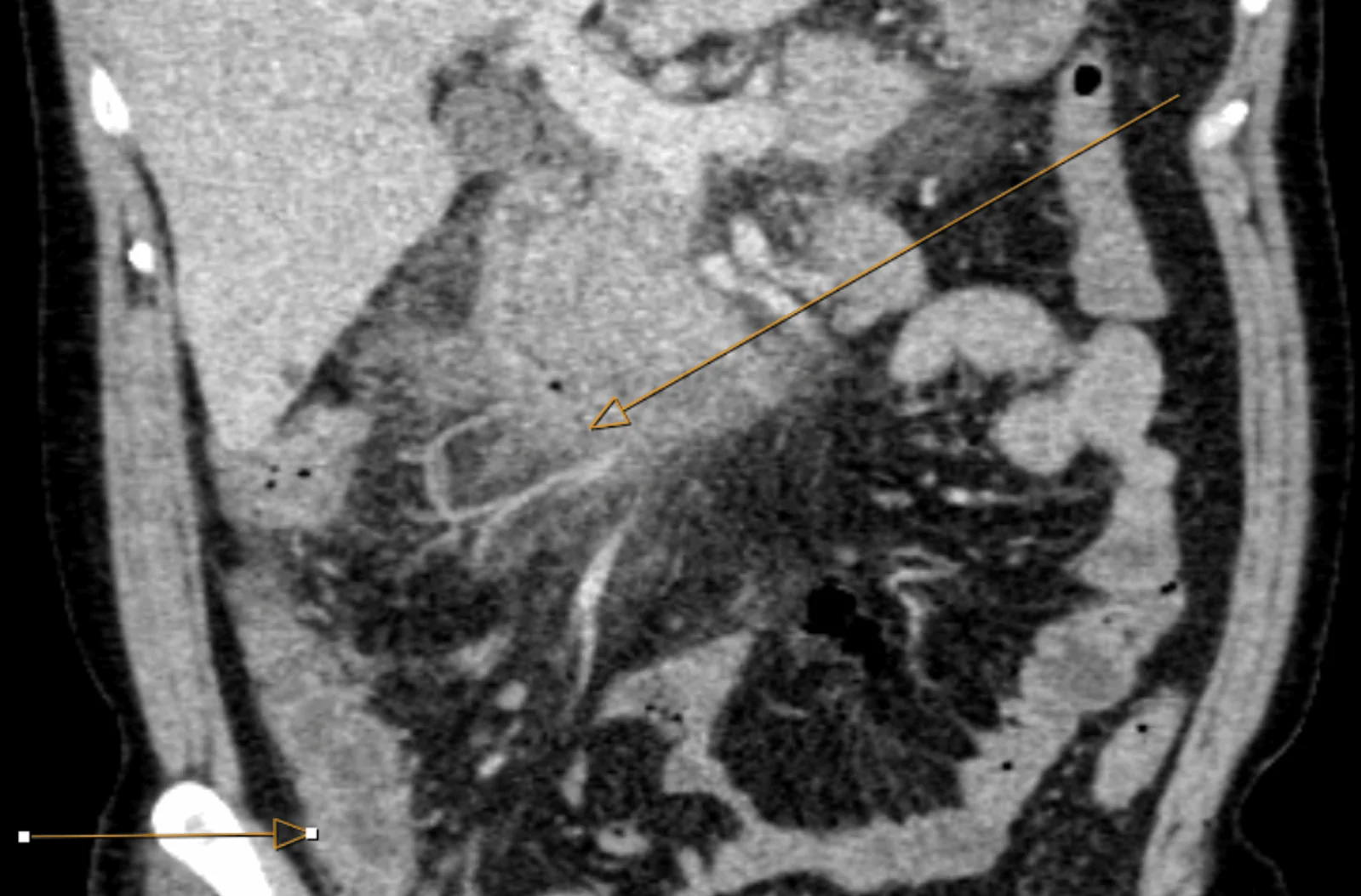
Repeat coronal contrast-enhanced CT of the abdomen and pelvis showing new wall thickening of the ascending colon, consistent with mild colitis (bottom arrow), and persistent pancreatitis (top arrow)

Therefore, repeat CT of the abdomen and pelvis was performed to assess for pancreatic complications, such as pancreatic abscess and necrotizing pancreatitis. This demonstrated more prominent extensive peripancreatic inflammatory fat stranding compared with the previous CT, along with new-onset colitis (Figure 4).

It was not until the fourth day of admission that a possible association between pancreatitis and acute gastroenteritis was considered, with *Salmonella *identified as the most likely causative organism. This suspicion was discussed with the microbiology team, who recommended awaiting the stool sample results. *Salmonella *was initially suspected because of its prevalence in North Africa and its documented ability to cause self-limiting gastroenteritis to overwhelming sepsis [7].

Despite the negative blood culture and pending stool sample results, and given the clinical suspicion of *Salmonella*-induced pancreatitis, empirical treatment with the third-generation cephalosporin ceftriaxone was initiated in accordance with CDC guidelines [8], along with metronidazole for empirical coverage of colitis.

On Day 11 of hospitalization, fecal PCR was positive for *Salmonella*, which was subsequently identified as *Salmonella typhimurium* subspecies type I. No antimicrobial susceptibility data were provided in the final report. The patient completed an eight-day course of intravenous ceftriaxone and metronidazole. No oral step-down therapy was required given his biochemical and clinical response.

The patient showed clinical improvement with supportive management and antibiotic therapy and was discharged on multivitamins alone. Laboratory investigations at discharge showed improving inflammatory markers, with resolution of abdominal pain and reduced stool frequency. He has had no further hospital admissions.

## Discussion

In our case, the initial working diagnosis on admission was acute infectious gastroenteritis based on the patient’s symptoms of diarrhea, abdominal pain, and fever, along with his recent travel history. Therefore, he was treated conservatively.

Given his recent return from North Africa and the presence of a new fever, further investigation, including testing for malaria, would ideally have been undertaken to exclude other potential causes of fever. However, in the absence of other clinical signs or symptoms suggestive of malaria, and given the overall clinical presentation consistent with acute gastroenteritis, malaria testing was not initially performed. This decision was further supported by the fact that the patient had travelled to areas that are not considered malaria-endemic. However, we acknowledge that this should have been considered in the diagnostic assessment on admission.

When the initial CT confirmed acute pancreatitis, alongside the clinical symptoms and biochemical markers, the working diagnosis was adapted to acute pancreatitis due to possible *Salmonella *infection. *Salmonella *is a common cause of gastroenteritis following recent travel, also known as travelers' diarrhea. Therefore, intravenous ceftriaxone and metronidazole were initiated.

Further imaging and blood tests were carried out to exclude other common etiologies of pancreatitis, but despite the extensive workup, the cause remained unclear. Following the initiation of ceftriaxone, the patient’s clinical condition improved, along with improvement in laboratory markers. This further strengthened our theory that pancreatitis was most likely a complication of *Salmonella *gastroenteritis.

Acute pancreatitis has a variety of etiologies, with the most common causes being gallstones and alcohol [3]. The suspected etiology in the case presented was infectious, which is uncommon and remains poorly understood.

Several mechanisms have been proposed to explain the pathophysiological relationship between *Salmonella *infection and acute pancreatitis. These include direct dissemination of the infection via lymphatic or hematogenous routes, as well as direct pancreatic involvement through migration of *Salmonella *from the duodenum and biliary tree into the pancreatic duct [3]. Additionally, other proposed bacterial mechanisms include the bile duct-pancreatic duct common pathway theory, pancreatic autodigestion theory, enzyme activation theory, microcirculation disturbance theory, and excessive leukocyte activation theory [3].

Viral infections have also been documented to cause pancreatitis as a complication. Similarly, the pathophysiological relationship between viral infection and pancreatitis is not fully understood.

Yu et al. have proposed that viral infection may directly injure pancreatic acinar cells through angiotensin-converting enzyme 2 (ACE2)-mediated entry, impairing exocytosis and promoting intracellular zymogen retention, with premature enzyme activation. Another proposed theory involves infection-induced cytokines. These cytokines may increase reactive oxygen species (ROS) production, resulting in oxidative stress and further cell injury [9].

*Salmonella enterica *is a significant cause of foodborne illness worldwide and is typically transmitted through the ingestion of contaminated food or water [4,10]. Acute infectious gastroenteritis caused by *S. enterica *can be broadly classified into typhoidal and nontyphoidal disease based on the causative serovar and clinical presentation. Typhoidal *Salmonella *has humans as its only reservoir, whereas nontyphoidal *Salmonella *affects a wide range of animals and humans [4,10,11].

Nontyphoidal *Salmonella *is usually a self-limiting disease, with features of acute gastroenteritis including fever, nausea, vomiting, and diarrhea. Among immunocompetent hosts, severe or invasive disease is infrequent, and most cases do not require antibiotic therapy. However, typhoidal *Salmonella *presents with a typical enteric fever picture, including fever, chills, and abdominal pain, with systemic complications such as hepatitis, pneumonia, and anemia that can affect multiple organs. It often requires prompt initiation of antibiotics to reduce complications [4,8,10,11].

The identified *Salmonella *species in this case, *S. enterica *serovar Typhimurium (nontyphoidal), generally causes self-limiting gastroenteritis; however, it can develop into a more serious condition if it spreads. It can cause distant organ infections such as cholangitis and pneumonia, although these usually occur in immunocompromised hosts. Treatment often involves conservative management, but antibiotics are given in severe cases, such as in immunocompromised patients, hospitalized patients, patients younger than 12 months or older than 50 years, those with severe diarrhea or persistent fever, and those with endovascular material or joint prostheses. The choice of antibiotics includes third-generation cephalosporins such as ceftriaxone [8].

There are a limited number of cases that have reported acute pancreatitis as a complication of *Salmonella *gastroenteritis, but there is no clear evidence proving causality in these cases (Table 2) [6,12-16].

**Table 2:** Summary of published case reports of Salmonella-associated pancreatitis

Published year	Clinical signs	Bacteria isolated	Treatment
2003	Diarrhea, nausea, vomiting, ecchymosis over umbilical area	Salmonella typhimurium	Ciprofloxacin + surgery - pancreatectomy
2016	Fever, vomiting, diarrhea, abdominal pain	*Salmonella enteritidis*	Third-generation cephalosporin
2018	Fever, vomiting, diarrhea, abdominal pain	*Salmonella *type D	Cefotaxime + metronidazole
2021	Diarrhea, vomiting, abdominal pain, fever	Salmonella typhi	Ceftriaxone
2021	Fever, vomiting, diarrhea, abdominal pain	*Salmonella *Group B	Piperacillin-tazobactam
2023	Diarrhea, vomiting, abdominal pain	Salmonella enteritis	Azithromycin

One case reported *Salmonella *infection associated with necrotizing pancreatitis, while another reported the coexistence of acute pancreatitis and acute hepatitis as complications of Salmonella infection [15,16].

The diagnosis of pancreatitis in the setting of ongoing *Salmonella *infection can be challenging due to overlapping clinical symptoms, in addition to false-positive elevations of biomarkers, particularly serum amylase, which may be considered biochemical pancreatitis rather than clinical pancreatitis. Hyperamylasemia has been reported in enteric infections, and several mechanisms have been proposed to account for hyperamylasemia in these infections. These include disruption of the intestinal mucosal barrier, which may increase intestinal permeability and facilitate the translocation and systemic absorption of pancreatic enzymes. Alternatively, impaired renal clearance may contribute to elevated serum amylase levels, particularly in the setting of renal dysfunction. Severe metabolic disturbances may also contribute to elevated serum amylase levels [17,18]. Several studies have demonstrated no clear association between elevated pancreatic enzymes during gastroenteritis and the presence of pancreatitis [17,18]. These findings highlight the importance of integrating clinical judgment with biochemical and radiological findings when establishing a diagnosis of pancreatitis in the setting of ongoing gastroenteritis.

In this case, given the clinical picture, recurrent fever despite early antibiotic initiation, and CT confirmation of colitis and pancreatitis, the patient may be considered to have had an invasive infection, as *Salmonella *infections are usually self-limiting. Invasive infection is common in immunocompromised individuals [19]; however, in this case, there was no history of an underlying autoimmune condition, malignancy, or immunosuppressive therapy. In addition, Lynch syndrome does not inherently increase the risk of salmonellosis. This suggests that invasive *Salmonella *infections may still occur in individuals who are not typically at risk, supporting the need for vigilant diagnosis and early treatment of possible invasive disease.

However, a key limitation of this report is the inability to establish a definitive causal relationship between *Salmonella *infection and the development of acute pancreatitis. Although the temporal sequence of gastrointestinal illness preceding pancreatic inflammation, together with the exclusion of more common causes of pancreatitis, supports a potential causal association, this alone cannot prove causality. However, these findings also do not exclude it.

The number of published case reports illustrating pancreatitis as a complication of *Salmonella *infection supports an association between these two diseases. The available evidence remains largely observational, and the precise pathophysiological mechanism of pancreatic injury is not clearly understood. Therefore, this case should be interpreted as contributing to the growing evidence supporting an association between *Salmonella *infection and acute pancreatitis.

## Conclusions

A diagnosis of possible acute pancreatitis due to *Salmonella *was made following careful exclusion of other common etiologies through radiological and biochemical investigations, as well as relevant patient history and examination. This diagnosis was further supported by the timeline of the patient’s symptoms and clinical improvement following the initiation of ceftriaxone and conservative management with intravenous fluids and antiemetics.

This case reinforces the importance of considering not only common but also rare potential etiologies of pancreatitis that may not be immediately apparent. *Salmonella *is one of the most common causes of gastroenteritis among returning travelers; therefore, it should always be considered in patients with a history of recent travel. Early recognition of such atypical presentations can reduce the risk of misdiagnosis and prevent delays in appropriate treatment.
